# Adapting a Theory-Informed Intervention to Help Young Adult Couples Cope With Reproductive and Sexual Concerns After Cancer

**DOI:** 10.3389/fpsyg.2022.813548

**Published:** 2022-02-04

**Authors:** Jessica R. Gorman, Karen S. Lyons, Jennifer Barsky Reese, Chiara Acquati, Ellie Smith, Julia H. Drizin, John M. Salsman, Lisa M. Flexner, Brandon Hayes-Lattin, S. Marie Harvey

**Affiliations:** ^1^School of Social and Behavioral Health Sciences, College of Public Health and Human Sciences, Oregon State University, Corvallis, OR, United States; ^2^Connell School of Nursing, Boston College, Chestnut Hill, MA, United States; ^3^Cancer Prevention and Control Program, Fox Chase Cancer Center, Philadelphia, PA, United States; ^4^Graduate College of Social Work, University of Houston, Houston, TX, United States; ^5^Department of Clinical Sciences, College of Medicine, University of Houston, Houston, TX, United States; ^6^Department of Health Disparities Research, The UT MD Anderson Cancer Center, Houston, TX, United States; ^7^Department of Social Sciences and Health Policy, Wake Forest School of Medicine, Winston-Salem, NC, United States; ^8^Wake Forest Baptist Comprehensive Cancer Center, Winston-Salem, NC, United States; ^9^Doctor of Physical Therapy Program, Oregon State University, Bend, OR, United States; ^10^School of Medicine, OHSU Knight Cancer Institute, Oregon Health and Sciences University, Portland, OR, United States

**Keywords:** young adult, cancer, sexual health, reproductive health, survivorship, sexual and gender minorities, qualitative, adaptation

## Abstract

**Objective:**

Most young adults diagnosed with breast or gynecologic cancers experience adverse reproductive or sexual health (RSH) outcomes due to cancer and its treatment. However, evidence-based interventions that specifically address the RSH concerns of young adult and/or LGBTQ+ survivor couples are lacking. Our goal is to develop a feasible and acceptable couple-based intervention to reduce reproductive and sexual distress experience by young adult breast and gynecologic cancer survivor couples with diverse backgrounds.

**Methods:**

We systematically adapted an empirically supported, theoretically grounded couple-based intervention to address the RSH concerns of young couples coping with breast or gynecologic cancer through integration of stakeholder perspectives. We interviewed 11 couples (22 individuals) with a history of breast or gynecologic cancer to review and pretest intervention materials. Three of these couples were invited to review and comment on intervention modifications. Content experts in RSH and dyadic coping, clinicians, and community advisors (one heterosexual couple and one LGBTQ+ couple, both with cancer history) participated throughout the adaptation process.

**Results:**

Findings confirmed the need for an online, couple-based intervention to support young couples experiencing RSH concerns after breast or gynecologic cancer. Qualitative themes suggested intervention preferences for: (1) A highly flexible intervention that can be tailored to couples’ specific RSH concerns; (2) Active steps to help members of a dyad “get on the same page” in their relationship and family building plans; (3) A specific focus on raising partners’ awareness about how cancer can affect body image and physical intimacy; and (4) Accessible, evidence-based information about RSH for both partners. These results, along with feedback from stakeholders, informed adaptation and finalization of the intervention content and format. The resulting virtual intervention, *Opening the Conversation*, includes five weekly sessions offering training to couples in communication and dyadic coping skills for addressing RSH concerns.

**Conclusion:**

The systematic adaptation process yielded a theory-informed intervention for young adult couples facing breast and gynecological cancers, which will be evaluated in a randomized controlled trial. The long-term goal is to implement and disseminate *Opening the Conversation* broadly to reach young adult couples with diverse backgrounds who are experiencing RSH concerns in cancer survivorship.

## Introduction

Young adult survivors of breast and gynecologic cancer (defined as those diagnosed between the ages of 18 and 39) face several unique challenges, including abrupt and often unexpected changes to their life plans and intimate relationships (Gorman et al., 2011, 2012, 2020). Younger survivors are at greater risk of psychological distress, as compared to those diagnosed at older ages (Arndt et al., 2004; Bidstrup et al., 2015; Acquati and Kayser, 2019). At least half of young survivors experience negative effects of cancer and cancer treatment on their reproductive and sexual health (RSH; Fobair et al., 2006; Wettergren et al., 2017; Jing et al., 2019). Adverse late effects of cancer on RSH include infertility, worry about personal health after pregnancy, concerns about potential risks to a future child’s health, hot flashes, poor body image, sexual pain, low sexual desire, concerns about disclosure to new partners, and related issues (Walshe et al., 2006; Karabulut and Erci, 2009; Carter et al., 2010; Grover et al., 2012; Robinson et al., 2014; Schover et al., 2014; Bradford et al., 2015; Wettergren et al., 2017). RSH concerns are among the most distressing aspects of life after cancer for young survivors and their partners, and when left unaddressed, often lead to poorer mental health and quality of life (Carter et al., 2010; Levin et al., 2010; Vaz et al., 2011; Canada and Schover, 2012; Robertson et al., 2016; Ljungman et al., 2018; Patterson et al., 2020). Despite the common and distressing nature of RSH concerns for many young adult survivor couples, these concerns are generally not adequately addressed by their healthcare providers (Gorman et al., 2021). Furthermore, there are no evidence-based interventions designed to help both young adult survivors and their partners reduce cancer-related reproductive and sexual distress. Therefore, development of age-specific interventions that support couples experiencing RSH concerns is essential.

There are several important considerations when developing an RSH intervention for young adult survivor couples. First, it is important to acknowledge that RSH concerns can be challenging to articulate because they encompass a variety of interwoven aspects (e.g., problems with sexual function alongside the desire for a biological child) and evolve over time along with the relationship, health status, and other life circumstances. Second, available approaches emphasize specialist care, such as sex therapists or fertility specialists, and are limited in scope, often focusing only on the survivor’s experience and neglecting support for partners. Third, these services are not widely available, particularly in rural areas, and access to care remains a barrier. Additionally, where psychosocial interventions are available, additional barriers to participation include time and travel requirements (Fredman et al., 2009; Regan et al., 2012). Finally, most interventions have been developed for heterosexual couples and have overlooked the needs and preferences of LGBTQ+ couples, who experience inequities in care and have significant unmet survivorship care needs (Boehmer et al., 2013; Hulbert-Williams et al., 2017; Seay et al., 2018). Although there is insufficient research on the RSH concerns of LGBTQ+ cancer survivors and partners, emerging literature points to a long-term impact on relationships and sexual intimacy, psychological distress, and the need for support for both partners (Kamen et al., 2015; Brown and McElroy, 2018; Kent et al., 2019). Additionally, there may be differences in the RSH experiences and needs of LGBTQ+ couples, but relationship factors influencing sexual satisfaction appear similar across groups and include sexual communication (Henderson et al., 2009; Fleishman et al., 2020).

Cancer is characterized as a “we-disease,” where couples navigate the experience together as a unit (Kayser et al., 2007; Lyons and Lee, 2018). Effective communication and dyadic coping, which encompasses the range of actions by one or both partners to cope with stressors and individual/joint strategies to assist the other partner with managing stressful situations or events, are important for psychosocial adjustment and relationship functioning for couples facing cancer (Regan et al., 2012; Badr and Krebs, 2013; Traa et al., 2015; Kayser et al., 2018; Acquati and Kayser, 2019). Although limited research on couple communication about reproductive concerns after cancer exists, evidence to date suggests that couple communication about fertility is important and beneficial to coping with these concerns, but it is sometimes avoided because of fears about partner discomfort, relationship problems, lack of understanding, and related concerns about the way infertility could impact the relationship (Hawkey et al., 2021a,b). There is also some evidence that couples with fertility concerns experience fear of abandonment and relationship difficulties (Dryden et al., 2014; Lehmann et al., 2018), suggesting that engaging both partners is important.

Couple communication is increasingly recognized as an important predictor of relationship functioning, sexual health, and both patient and caregiver outcomes (Badr, 2017; Otto et al., 2021). In general, engaging both partners is important because couples who communicate effectively and engage in joint coping efforts have more positive relationship and mental health outcomes (Manne et al., 2006, 2015; Badr et al., 2008; Lyons et al., 2014, 2016). Conversely, when partners withdraw, engage in protective buffering, or hold back, poorer outcomes have been documented (Manne et al., 2006, 2015; Badr et al., 2008; Manne and Badr, 2008). With regard to sexual health, partners’ active engagement in coping with patients’ cancer-related sexual concerns is important because (a) sexual concerns are often experienced in the context of partnered sexual activity (Ganz et al., 2002; Fobair and Spiegel, 2009), (b) partners commonly report sexual health concerns (Loaring et al., 2015; Hummel et al., 2017a), and (c) survivors tend to want their partners involved in this process (Reese et al., 2016). Indeed, the most effective approaches to addressing sexual health and reducing sexual distress after cancer have systematically engaged partners (Schover et al., 2013; Carroll et al., 2016; Hummel et al., 2017b). In sum, it follows that couple-based interventions to improve dyadic coping strategies and effective communication represent a promising strategy for improving the relationship functioning of couples coping with the long-term effects of cancer on their relationship and that these approaches may be effective for reducing RSH-related distress across the cancer continuum (Scott and Kayser, 2009; Hawkey et al., 2021a).

Evidence indicates that psychosocial interventions enhance dyadic coping and communication in the context of cancer (Badr and Krebs, 2013; Traa et al., 2015; Li et al., 2020) and that they may be most effective for improving sexual health and quality of life when incorporating elements of psychoeducation, skills training, and couple-counseling (Li et al., 2020). One such intervention, *Side by Side*, provides training for individual and relationship skills specific to breast and gynecologic cancer survivor couples’ experience. It focuses heavily on sharing thoughts and feelings and couple communication about cancer-related issues. The intervention was designed for delivery *via* four in-person sessions of 2 h each. In a randomized controlled trial involving 72 heterosexual German couples (age 25–80 years, median age 52 years) who were married or in a committed relationship, those in the active condition reported less avoidance in dealing with cancer, more posttraumatic growth, better communication quality, and better dyadic coping than those in an attention control condition (Heinrichs et al., 2012).

*Side by Side* is grounded in methods of cognitive behavioral therapy (Epstein and Baucom, 2002) and Bodenmann’s conceptualization of dyadic coping (Bodenmann and Shantinath, 2004; Bodenmann et al., 2006; Heinrichs et al., 2012). It was originally based on CanCOPE (Scott et al., 2004) and was previously modified in a pilot trial (Zimmermann et al., 2006). Following Bodenmann’s Systemic Transactional Model (STM), one partner’s stress appraisal influences and it is influenced by the other partner and the relationship (Bodenmann, 1995, 1997; Bodenmann et al., 2016). Following this theory, dyadic coping mitigates the negative impact of stress on a couple’s relationship (Bodenmann, 1997, 2008; Bodenmann and Shantinath, 2004). Dyadic coping involves cognitive (e.g., stress appraisal), emotional (e.g., shared emotions), and behavioral processes (e.g., active listening and problem solving) where both members of the couple participate as equal partners. To enhance dyadic coping, *Side by Side* incorporates training and practice in Bodenmann’s three-phase method (Bodenmann, 2007). The three-phase method helps partners to: (1) communicate their stress to their partner, (2) meet the specific needs of the stressed partner, and (3) improve their ability to cope together with the stress. Following the STM, stressors can include daily life stressors or more severe stressors, such as those resulting from illness (Bodenmann, 2007; Bodenmann et al., 2016). The present study extends application of the theory to stressors related to cancer’s impact on RSH in young adult couples, which can range in form and severity.

In the present study, we describe the systematic adaptation and tailoring of *Side by Side* for young adult couples with breast or gynecologic cancers, who are 6 months to 5 year post-diagnosis, and with any sexual orientation or gender identity, to help them communicate about and cope with RSH concerns. The primary outcomes are sexual and reproductive distress. The intervention format was also adapted for videoconference delivery. Our overarching goal was to optimize acceptability and feasibility while retaining core components (i.e., intervention practices linked to theory-driven mechanisms of change). Our specific goals for the adaptation process were to increase fit/relevance, elicit and address the primary RSH-related concerns for both survivors and partners, and increase LGBTQ+ inclusivity. This work was completed in preparation for the intervention’s efficacy testing *via* randomized controlled trial (NCT04806724).

## Materials and Methods

**Figure 1:**
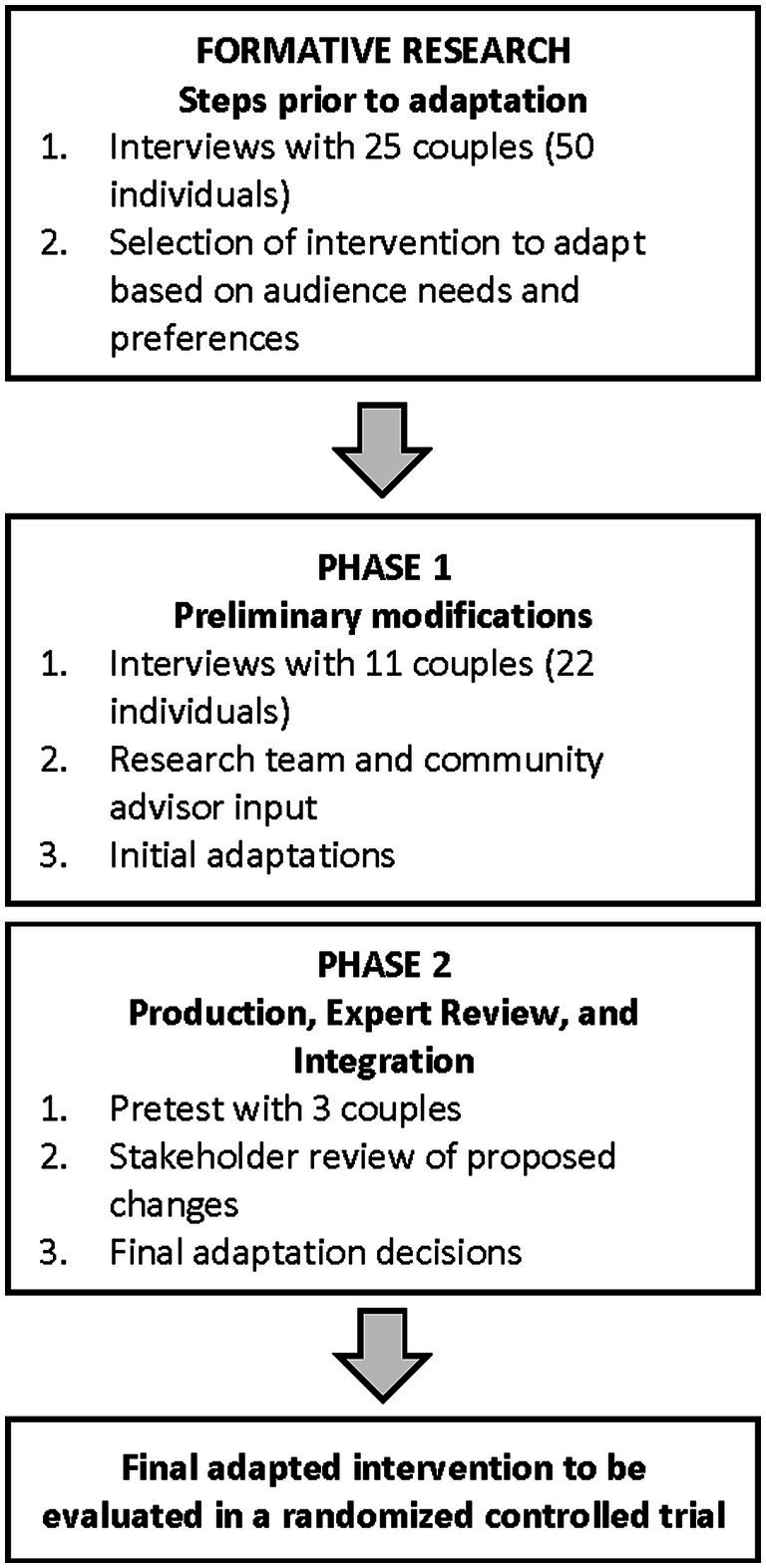
Summary of intervention adaptation process. Formative research results are published elsewhere.

We adapted the intervention following the assessment, decision, adaptation, production, topical experts, integration, training, and testing (ADAPT-ITT) framework’s systematic process (McKleroy et al., 2006; Wingood and DiClemente, 2008), including integration of target audience and other stakeholder perspectives (Figure 1). ADAPT-ITT has evolved over years of work by the CDC and others in the context of HIV, has resulted in successful and cost-effective intervention adaptations, and follows commonly recognized steps in the adaptation process (Wingood and DiClemente, 2008; Latham et al., 2010; Escoffery et al., 2019). Importantly, systematic adaptation facilitates intervention fit for a specific audience and setting while retaining core components of the intervention, which is essential for future implementation and dissemination (Escoffery et al., 2019).

Formative research conducted prior to the present study involved individual interviews with 25 young adult survivor dyads (50 individuals) to assess psychosocial supportive needs and to identify an intervention strategy that would meet that need (Gorman et al., 2020). Importantly, couples reported that maintaining open communication was central to preserving strong relationship functioning through cancer and expressed preferences for a couple-focused intervention strategy providing support for both partners that was delivered in an online/virtual format (Gorman et al., 2020). This formative research was guided by the theory of dyadic illness management, which posits that the way couples experience cancer is influenced by each partner’s appraisal and management of its impact and that this ultimately affects the health of both partners (dyadic health; Lyons and Lee, 2018). Our findings revealed that couples experience a wide range of RSH concerns and that these vary according to contextual factors, such as current life circumstances. Results also highlighted the promise of intervention strategies facilitating “togetherness,” mutual support, and collaborative management of RSH concerns after cancer to support dyadic health, further building upon the theoretical foundation for the adapted intervention (Gorman et al., 2020). Based on our results, and a review of the literature validating substantial need for intervention focused on RSH for this population (Stabile et al., 2017; Logan et al., 2018, 2019; Jing et al., 2019), we consulted with the intervention developers and selected *Side by Side* for adaptation. This dyadic intervention was selected based on a combination of factors suggesting fit for the audience and evidence for potential impact, including alignment with needs and preferences identified in our formative research, targeted focus on communication and coping skills, grounding in theory and evidence-based practice, specificity for breast and gynecologic cancer survivors, and demonstrated effectiveness for improving relationship and psychosocial outcomes (Epstein and Baucom, 2002; Bodenmann et al., 2006; Heinrichs et al., 2012).

A stakeholder panel was assembled that included content experts in RSH concerns after cancer, dyadic interventions, dyadic coping behaviors and illness management after cancer, AYA survivorship, AYA oncology, oncology social work, and four community advisors, one heterosexual couple, and one LGBTQ+ couple, both with a cancer history and representing diversity in sexual orientation as well as trans/cisgender identity. We purposefully invited stakeholders representing diverse perspectives who were familiar with or identified as members of the intended audience for the intervention. We also sought stakeholders with medical care roles as well as social/psychological care roles. There were not specific inclusion or exclusion criteria.

The study protocol was approved by the Oregon State University Institutional Review Board. All study participants completed oral informed consent procedures.

### Phase 1: Administration-Preliminary Adaptation and Feasibility Assessment

The purpose of this step was to consult with the intended audience to determine factors that would support RSH communication after cancer and to review *Side by Side* intervention materials with the goal of eliciting stakeholders’ perspectives on content adaptation. We used purposive sampling to represent and reflect the diversity of perspectives in the intended audience and to document potential variations and common patterns (e.g., needs and preferences) that cut across cases to inform intervention adaptations that meet diverse needs (Palinkas et al., 2015). Eligibility criteria for cancer survivors were: breast/gynecologic cancer diagnosis between the ages of 18 and 39 years, current age under 45 years, cancer diagnosis 6 months–5 years prior, cancer stage 1–4, moderate or higher reproductive concerns in one domain of the Reproductive Concerns After Cancer scale (Gorman et al., 2014), ability to participate in a Zoom interview, a committed partner who is willing to participate, English speaking, and high speed Internet access. Inclusion criteria for partners were: age 18 or older, English speaking, ability to participate in a Zoom interview, and high speed Internet access.

#### Data Collection

Trained team members conducted individual semi-structured interviews *via* Zoom (audio only) with each partner separately. Interviews were completed between August 2020 and January 2021. The semi-structured format provided an opportunity to explore ideas and clarify concepts throughout the interview (24). Participants were provided with a bulleted summary of *Side by Side* intervention content/activities and sample participant handouts prior to the interview. Interviews were approximately 1 h in duration and were audio recorded and professionally transcribed. The interviewer first asked questions about demographics, cancer history, and reproductive history. Interview questions covered the following domains: general relationship (e.g., “How would you describe your relationship with your partner?”), communication about sexual health (e.g., “How do you keep your communication open about sexual health?” and “How did you talk about sex before your/your partners’ cancer diagnosis?”), communication about reproductive health (e.g., “How has your/your partner’s experience with cancer affected how you talk about fertility or having children?” and “How often do you talk to your partner about fertility or having children?”), preferred aspects of the intervention (e.g., “Thinking about what would help you and your partner, what are the most important issues or skills that you think the program should focus on?”), intervention content to address RSH specifically, while reviewing the *Side by Side* materials provided (e.g., “What kinds of resources and information would you like to see included in the program to help you to better manage your reproductive or sexual health after cancer?”), and intervention preferences related to feasibility and acceptability (e.g., “How much time per week could you imagine yourself being able to commit to this program?”, “Please describe the person you would feel most comfortable leading the weekly sessions” and “What are your thoughts on having this program delivered online, by videoconference (for example, Zoom) where a counselor would connect privately with you and your partner in your home?”).

#### Analysis

Transcripts were imported, organized, and coded in QSR NVivo 12 software. Interviews were analyzed utilizing a structural coding approach (Saldaña, 2015). The initial codebook was developed based on interview guide domains. Then, two members of the research team completed initial coding of survivor and partner interviews separately to determine the final codebook. Once a codebook was developed, we moved to structured coding, where we identified the most relevant and common codes and applied them to the transcripts. Next, researchers employed thematic analysis to identify themes and patterns in the data that informed intervention adaptations (Braun and Clarke, 2006). Researchers met frequently during the coding process to discuss findings and employed memo-taking to increase reliability of results. Results were compared between survivors and partners to identify potential differences in responses or preferences and then summarized and subsequently reviewed by members of the research team. The team also consulted with the community advisors who reviewed materials and gave feedback on planned adaptations during this phase. The research team came to consensus on final themes and decisions about adaptations.

### Phase 2: Production, Expert Review, and Integration-Development of the Final Adapted Intervention

The research team first translated feedback obtained in the prior step to inform modifications to the intervention content and format. Adaptations focused on improving relevance to the target population, addressing the RSH-related concerns of survivors and their partners, aligning with intended audience expressed needs, and modifications for a virtual intervention setting. To develop new educational content focused on RSH concerns, the team conducted a review of the literature to identify potential adverse effects of cancer on RSH (e.g., changes in fertility and sexual function) and recommended strategies to address those concerns (e.g., discussion with a fertility specialist and use of vaginal health aids). Adaptation decisions were agreed upon by five team members after each round of revisions. The team took care to maintain all intervention practices linked to theory-driven mechanisms of change (e.g., speaker/listener skills, Bodenmann’s three-phase-method training and practice, and coping self-talk). After making these modifications, three couples of diverse sexual/gender orientations were invited back to review and comment on the resulting participant materials.

#### Data Collection

Couples received a copy of the revised and new participant handouts and a bulleted summary of the weekly intervention topics/activities to review prior to their interview. Couple interviews were conducted together *via* Zoom, following a semi-structured interview guide. Interviews were approximately 60–90 min in duration and were audio recorded. Participants were first asked questions about each of the five sessions (e.g., “Was there anything you did not understand?,” “What aspects do you think would be most helpful to you?,” “Are there aspects of the handouts that we should change related to reproductive or sexual health?,” and “What other suggestions do you have for improving the materials?”). This was followed by a series of questions about the overall intervention, including general comments about the flow of the intervention/sessions, things they particularly liked or disliked, aspects they would include or change, and ways we could bring the program to the attention of couples who might benefit.

#### Analysis

Interviewers documented responses and reviewed transcripts to categorize recommendations, which were reviewed and discussed by the research team to inform further adaptations. Similar to structural coding procedures, interviewers developed categories for recommended changes based on interview guide questions (Saldaña, 2015). Responses related to potential adaptations were listed and categorized. The few that did not align with the scope of research or were not feasible were not implemented (e.g., adding elements focused on diet and exercise).

To obtain an assessment of consensus on adaptation decisions, the resulting revised materials were then disseminated to all stakeholders, including community advisors, who were asked to provide feedback *via* an online survey. Prior to completing the survey, stakeholders were asked to review the participant handouts and modifications to the interventionist manual focusing on content related to RSH. The research team made final modifications based on this feedback. Intervention adaptations were tracked and summarized (Wiltsey Stirman et al., 2019).

## Results

### Phase 1: Administration-Preliminary Adaptation and Feasibility Assessment

In this first phase, 11 couples participated (22 individuals) in separate qualitative interviews. This included eight breast cancer and three gynecologic survivors (two ovarian and one cervical), one survivor who identified a gay/lesbian, one survivor who identified as bisexual, and one partner who identified as gay/lesbian. Survivors ranged from ages 29 to 40 years (*M* = 35.2 years) and were diagnosed between 6 months and 6 years ago (*M* = 3.0 years since diagnosis). Partners were ages 25–42 years (*M* = 34.6 years). Relationship duration varied from 1 to 22 years (*M* = 10.2 years; Table 1).

**Table 1:** Participant characteristics.

	Cancer Survivors (*N* = 11) *n* (%)	Partners (*N* = 11) *n* (%)
**Demographic Characteristics**		
Current Age, yrs^a^	35.2 (3.7)	34.6 (5.7)
Race		
Asian	1 (9)	1 (9)
Native Hawaiian/Pacific Islander	0 (0)	1 (9)
White	10 (91)	9 (82)
Hispanic/Latinx	0 (0)	0 (0)
Gender		
Man	0 (0)	10 (91)
Woman	11 (100)	1 (9)
Sexual Orientation		
Bisexual	1 (9)	0 (0)
Gay/Lesbian	1 (9)	1 (9)
Heterosexual	9 (82)	10 (91)
Married	9 (82)	9 (82)
Relationship duration, yrs^a^	10.2 (5.5)	10.4 (4.6)
College graduate	10 (91)	8 (73)
Employed	8 (73)	9 (82)
**Cancer Characteristics**		
Type		
Breast	8 (73)	–
Cervical	1 (9)	–
Ovarian	2 (18)	–
Stage at diagnosis		
1	4 (36)	–
2	4 (36)	–
3	3 (28)	–
Age at diagnosis, yrs^a^	32.2 (3.0)	–
<35 years old	9 (81.8)	–
≥35 years old	2 (18.2)	–
Time since diagnosis, yrs^a^	3.0 (2.0)	–
**Reproductive Characteristics**		
1+ live births	4 (36)	–
Seen fertility specialist	5 (45)	4 (36)
Currently pregnant	1 (9)	0 (0)
Currently trying for pregnancy	0 (0)	0 (0)
Wants a/another baby	9 (82)	6 (55)
Sexually active	11 (100)	11 (100)
Uses contraception	5 (45)	4 (36)
Biological children important	5 (45)	6 (55)
Interested in adoption	5 (45)	5 (45)
Time since diagnosis, yrs^a^	2.5 (2.3)	**–**

a Mean (standard deviation).

The following themes emerged after Phase 1 interviews, suggesting intervention preferences for: (1) A highly flexible intervention that can be tailored to couples’ specific RSH concerns; (2) Active steps to help members of a dyad “get on the same page” in their relationship and family building plans; (3) A specific focus on raising partners’ awareness about how cancer can affect body image and physical intimacy; and (4) Accessible, evidence-based information about RSH for both partners. Results did not reveal any differences between the intervention preferences of survivors or their partners. Table 2 demonstrates how themes informed intervention adaptations.

**Table 2:** Translation of qualitative results to intervention adaptation.

Theme	Illustrative Quotes	Adaptations
*Theme 1. A highly flexible intervention that can be tailored to couples’ specific RSH concerns*	“So I think the counselor getting that picture could really illuminate the type of discussions that are going to be happening between that couple. I think that again, the counselor should explicitly get a picture of, from each person, what their goals are as far as their own fertility, like do they want kids, blah, blah, blah, like how important. That should be something that’s explicitly included.” “Maybe my one feedback would be, it’s going to take a lot of sessions, maybe like two or three, just to really understand the patient. My story is really long and complicated. It’s not just I had cancer. It’s like the whole story that gets you there, I think helps develop an understanding for where someone is at.” “I think maybe an important thing that could be included is an explicit inclusion of the counselor or whoever is running the session, they should get a picture of what the prognosis for that couple’s diagnosis, just so that they can understand, “Okay this is like game over for fertility,” or in my case it was like there’s a 10% chance that you are going to lose fertility.”	Discussion about couples’ history, needs, and specific RSH concerns Flexible prompts for RSH discussion topics across sessions New session 5 to allow them to practice supportive communication with RSH topic of choice Diverse range of RSH topics in new educational material
*Theme 2. Active steps to help members of a dyad “get on the same page” in their relationship and family building plans*	“The emphasis on the communication, I think is so important. I think I take our communication for granted, because I think it’s really, really strong. I think that’s the most important thing, is being able to communicate things that, I mean it is difficult to tell your partner, ‘I do not want to talk about this right now. It’s nothing you did.’ Or, ‘I do want to talk about this. Are you available?’” “So, finding a common ground to be together and support each other on is, I think, important. I think this event can make it difficult to see that or get there, and potentially having a program or an outside third party to bring that around could be good.” “And understanding my partner’s different than me, and the support that they need, and the support that I need. And I think being able to come to a common ground and understanding of one another and the needs of each other is huge.”	Opportunity for fertility/family building focused supportive communication practice in session and at home Educational material to support shared understanding
*Theme 3. A specific focus on raising partners’ awareness about how cancer can affect body image and physical intimacy*	“I think the physical intimacy, especially for men, I feel like that’s such a big part of it. The conversations and stuff, I think, are really important, but I also think that there is something to be said for that kind of physical connection. Again, whether it’s holding hands and going for a walk or something where it’s a physical connection because you do kind of feel… It’s super easy to feel just like a cancer patient and not like a human on some levels. I think that that… I know, especially in the beginning, my husband was like, ‘Can I touch you? Are you okay? I do not know what to do.’ And so it’s like establishing those here’s what my boundaries are and encouraging that, I think would be really helpful, too.” “I think that kind of emotional… talking about the self-esteem and changes to the body and how a partner can support the person going through cancer and struggling with that.” “Some of the things that I’ve mentioned, like how to navigate intimacy when you are dealing with physical changes to your body because of cancer treatment, how to have the discussions about fertility preservation, and then adjusting your life plans based on the reality of your cancer. Also, talking about birth control, that’s part of the conversation about, how can sex be pleasurable and rewarding for both parties after so much change…”	Exercise to promote shared understanding of perspectives on emotional and physical intimacy Educational material to support shared understanding Home practice focused on intimacy building activities
*Theme 4. Accessible, evidence-based information about RSH for both partners*	“I would say the most practical stuff you can give is the best. Like, ‘Here’s what happens to fertility and here are these… there’s this information about how these things are normal and how there are lots of support groups out there.’” “My friend also had her hysterectomy. And she’s like, ‘Oh, yeah, I went on Amazon, and I bought myself a vibrator.’ And it was like, way better than just this dilator that’s like this hard piece of plastic that you shove up inside you. And I was like, ‘Oh, God, I wish I would have known that.’ So maybe those types of resources.” “And I think it would just be nice to have at your fingertips some of those programs and aids and organizations that… All in one place. Because I just think it’s all so scattered. And again, what you do not know, you do not know. And so I think having a centralized database or information base would be really good to have.”	Educational material on RSH topics with bullet evidence-based information, options to consider, and tips for partners Trusted resource list of online resources on a range of RSH topics Tips for healthcare provider communication/patient advocacy Glossary of terms

#### Theme 1. A Highly Flexible Intervention That Can Be Tailored to Couples’-Specific RSH Concerns

Couples described a desire for an intervention that can “meet them where they are,” as opposed to one-size-fits-all approach, as it relates to their specific RSH concerns. One survivor explained, “So that would be my biggest comment; to design these programs or this program and sessions in a way that does not try to get all the cancer patients in the same pot, but differentiate between where, what kind of stage they are in and what kind of life stage and cancer stage they are in, I would say.” One way couples noted that this could be achieved is spending time in the first session getting to know the couple including their history with cancer, relationship duration, and stage of family building goals [e.g., already have child(ren) or not]. For example, one survivor described, “For me, I think what you’ll find is that every couple is kind of different. And every couple obviously is going to bring different things to the table. I mean, you are going to have couples that it’s their fifth kid, and they are in their later years, they were not planning on having any more, or you are going to have couples that did not get any kids.”

#### Theme 2. Active Steps to Help Members of a Dyad “Get on the Same Page” in Their Relationship and Family Building Plans

Couples explained a desire for the intervention to help them align their goals and priorities regarding their family building plans and intimate relationship. For example, several survivors noted that couples may not feel comfortable sharing intimate information with each other, especially in front of an interventionist, and that it is important to include an opportunity to hear both survivors’ and partners’ perspectives. One survivor noted this would be facilitated well by the interventionist during the session, “I just feel like I’m always trying to explain myself and I do not always do a great job of fully explaining, and so to have a very detailed professional person explaining what is going on makes a lot more sense.” Couples noted how this should be followed by learning “skills” to not only manage these difficult conversations but take active steps forward in their relationship. For example, one survivor described, “I think maybe, especially as things come up, let us say a fertility conversation comes up or something like that, kind of like a ‘next steps’, like a ‘where to go from here,’ because now we have got the foundation to be able to have these conversations, but like a ‘now what’ would be helpful.”

#### Theme 3. A Specific Focus on Raising Partners’ Awareness About How Cancer Can Affect Body Image and Physical Intimacy

Couples emphasized the importance of including activities and elements focused on helping the partner without cancer to understand physical body changes experienced after cancer and how those might impact their intimacy. For example, one survivor explained her thoughts and feelings in this way:

“I think what would be most important to me is particularly the physical changes… The physical changes that affect self-esteem and self-worth as a woman, if you will. It is not something that a man can necessarily always super relate to… I think that having conversations around and encouraging conversations around like, hey, it is okay that you feel this way. I still love you regardless. Kind of talking about more of the emotional impact of the cancer treatments as they change you physically.”

Survivors noted that the program should include skills for “how a partner can support their person going through cancer and struggling with that [body image],” as they also describe their challenges with body image, for example, feeling “ugly and sick and undesirable.” Several partners described a desire for skills around listening and processing difficult conversations. Survivors expressed a need to build their confidence through skill building, in addition to having conversations. One survivor stated, “I think it is expanding up this “ways to express tenderness and closeness” part… It needs to be more than just a conversation… there needs to be daily activities and strategies and exercises around being romantic again.”

#### Theme 4. Accessible, Evidence-Based Information About RSH for Both Partners

A leading request from couples was to include educational materials for both partners about how cancer can affect RSH. Couples reported a “huge lack of information for the patients and for their partners about anything” related to RSH after cancer. In addition to information provided during the intervention, couples desired “somewhere you can go back and find more information or more resources” once completing the intervention for sustained engagement and learning opportunities. As one survivor said as:

“If there were bullet points and checklists, those are things that if they boiled down some takeaways where if you have completed the program and you want to go back to it and were able to just go through bullet points and it would refresh you on various things.”

Couples wanted information and resources to be accessible, reliable, and educational, such as links to videos of other survivors sharing their experiences. They identified several types of information they would like to see included, such as how to manage health insurance related to family building needs, the range of emotional and physical changes couples experience after cancer, fertility preservation options, statistics on cancer treatment’s impacts on fertility, legalities of fertility preservation, and alternative family building options outside of biological parenthood. They also described the importance of gaining skills during the sessions that they could take home with them to continue having conversations after the intervention ended. One survivor said, “I think you need to add a whole session on the end that it is like a counselor facilitated discussion to get the conversation started between the two partners about intimacy and fertility. So that door is opened, and then they can go home and finish the conversation or continue to talk about it.” Thus, accessible RSH information was perceived as important for increasing shared knowledge and continued engagement and sustainment of behavior change after the intervention ended.

### Phase 2: Production, Expert Review, and Integration-Development of The Final Adapted Intervention

This phase informed development of the final adapted intervention. Table 3 summarizes intervention modifications across both phases. After changes from Phase 1 were integrated, the stakeholder panel reviewed and made minor additional edits based on prior expertise with dyadic intervention. Then, three couples were invited back to review and comment on the materials. Participants included one heterosexual, one bisexual, and one lesbian couple and both breast and gynecologic cancer survivors. Survivors ranged in age from 34 to 38 years old (*M* = 36.3 years) and were diagnosed between 6 months and 5 year prior (*M* = 2.5 years since diagnosis). Partners were between 25 and 39 years old (M = 32.7 years). Relationship duration for these couples ranged from 6 to 8 years (*M* = 7.3 years). Feedback from community advisors affirmed the changes made during Phase 1 and provided more specific feedback to further improve inclusivity for LGBTQ+ survivors (e.g., editing language to avoid any assumptions about sexual orientation or gender identity), adding minor elements (e.g., new medical terms to the glossary), and modifying design (e.g., more closely aligning the look of new and original handouts). The stakeholder panel supported the implementation of all proposed changes and provided additional minor edits to materials (e.g., wording recommendations to improve comprehension and identifying spelling errors).

**Table 3:** Summary of major intervention adaptions across phases.

Phase	Type of Modification	What was modified?
Initial adaptations based on Phase 1 interview results, research team expertise, and consultation with community advisors	Context	*Format/setting* Videoconference delivery *Audience* Young adult couples
	Content	*Tailoring* Inclusive language for LGBTQ+ couples *New content* Evidence-based information about RSH after cancer Reproductive health discussion/exercises Sexual health and body image discussion/exercises Patient advocacy and patient-provider communication Specific to partner/caregiver Specific to LGBTQ+ couples Discussion of options and action steps for RSH Trusted resources Glossary of terms *Removing content* Focus on immediate post-cancer timeframe *Tweaking/Refining* Reorganized session content Content on mindfulness Language for comprehension and usability Visual look of materials
Adaptations based on Phase 2 pretest	Content	*Tweaking/Refining* Specific to LGBTQ+ couples Emphasize flexibility to address RSH concerns More focus on partner perspectives Handout clarity, relevance, comprehension, and visual elements
Adaptations based on Phase 2 stakeholder review	Content	*Tweaking/Refining* Add glossary terms Handout clarity, relevance, comprehension, and visual elements Session flow

Modifications were made to increase fit/relevance, address the primary RSH-related concerns for both survivors and partners, and increase LGBTQ+ inclusivity, with the goal of optimizing feasibility and acceptability of *Opening the Conversation*. Key modifications included adding educational material for both partners to review and discuss focused on a range of RSH-related concerns (e.g., contraception, pelvic health, fertility, family building, and sexual health). When creating materials, the team considered participants’ desires for “digestible” information, but also of “hard data,” statistics, and potential solutions or next steps. Other key modifications focused on integrating opportunities to focus on RSH topics across all sessions in addition to adding a fifth session to provide an opportunity for couples to use skills learned during the intervention to focus on an RSH topic of their choice. New handout material included tips on a variety of topics that couples indicated a desire for, including patient advocacy, communicating with healthcare providers about RSH, tips specific to partners, use of lubricants and moisturizer, and LGBTQ+ specific resources. We also developed a trusted online multimedia resources list. Some original *Side by Side* content was removed, such as aspects focused on cancer’s immediate impact on their lives, to increase fit for the intended audience (younger age, 6-months to 5-years post-diagnosis). All fidelity/core elements (e.g., training in speaker/listener skills and three-phase method) were retained (Table 3).

The resulting intervention, *Opening the Conversation*, includes five weekly modules (1.5 h each) to be delivered *via* videoconference by a masters-level trained interventionist and organized around the following topics: (1) Understanding the impacts of cancer and ways to support one another; (2) Building coping and communication skills for both partners; (3) Practicing coping skills individually and together; (4) Sustaining a strong relationship after cancer: Emotional and physical intimacy; and (5) Sustaining a strong relationship after cancer: Reproductive health, family building, and relationship goals. To avoid a “one size fits all” approach, all participants receive RSH educational materials covering a wide range of topics and are encouraged to review and select those that are most relevant. Additionally, each session contains flexible discussion prompts, which allow couples to select discussion topics relevant to their unique situation and RSH concerns.

## Discussion and Conclusion

The iterative, systematic adaptation process yielded a theoretically grounded intervention, *Opening the Conversation*, which, if determined to be efficacious, could fill a critical gap in supportive care for young breast/gynecologic cancer survivors and their partners (Keesing et al., 2016; Gorman et al., 2021; Hawkey et al., 2021b) who are experiencing RSH concerns. Feedback at multiple time points and from diverse stakeholders guided decisions about intervention modifications. In sum, the results provided essential guidance for development of an inclusive, flexible psychosocial intervention for young couples facing a range of different RSH concerns after cancer that includes education and skill building opportunities to improve dyadic coping and communication.

Prior research has demonstrated the utility of dyadic interventions for enhancing relationship functioning after cancer (Manne and Badr, 2008), with emerging data also suggesting benefits for couples experiencing reproductive (Lehmann et al., 2019; Hawkey et al., 2021b) and sexual distress (Badr and Taylor, 2009; Reese et al., 2014; Perz et al., 2014; Reese et al., 2019; Gorman et al., 2020). A novel aspect of *Opening the Conversation* is the focus on both reproductive and sexual concerns, which couples often experience in tandem (Luk and Loke, 2019; Hawkey et al., 2021b). This represents a promising approach to supporting couples experiencing one or more RSH concern after cancer. Indeed, couples in this study stressed the importance of an intervention that would be flexible in the sense that it could be tailored to address their current RSH needs and concerns. Because RSH concerns and needs change over time (Gorman et al., 2021), couples can continue to benefit from knowledge and skills gained to address new issues as they arise. Based on our results, the flexibility to tailor to specific RSH concerns and inclusion of a broad range of RSH concerns are essential to help couples understand and address their RSH concerns as a unit.

An important finding in this study was that couples described the value of a chance to “name out loud” their RSH concerns but also wished to go beyond “opening the conversation” to build their skills and decide on “action steps” together. They specifically discussed this in two primary contexts. First, they talked about a wish to align their goals and priorities regarding family building. In other research, couples have also reported the benefits of open communication about fertility, along with several challenges including avoidance of discussion for fear of upsetting their partner (Benyamini et al., 2009; Hawkey et al., 2021a). *Opening the Conversation* provides an important opportunity for couples to share their perspectives and improve upon supportive communication to manage fertility-related concerns as a team. Similarly, most couples felt that discussions about sexual health and body image could be difficult to navigate and wished for knowledge and skills to help them do this. Numerous studies have demonstrated the challenges faced by couples in addressing sexual concerns after cancer (Hawkins et al., 2009; Gilbert et al., 2011; Robinson et al., 2014; Ussher et al., 2014; Dobinson et al., 2015; Loaring et al., 2015). Our results indicate that young couples desire and would benefit from intervention strategies that facilitate effective communication to enhance mutual understanding and management of sexual health challenges together. Overall, results suggest that improving the quality of communication for couples facing RSH concerns is an essential aspect of the intervention.

One particularly novel aspect of this study is that we centered LGBTQ+ identifying couples’ perspectives during the adaptation process, which informed inclusion of new materials (e.g., online resources and educational information) as well as use of inclusive language and content across sessions. LGBTQ+ survivors and their partners have significant unmet survivorship care needs specific to sexual health (Seay et al., 2018) where partners are in need of support, and the impact of cancer on relationships can be devastating (Brown and McElroy, 2018). LGBTQ+ individuals also often do not feel welcome in clinic/support group settings and experience poorer satisfaction with care than heterosexual survivors (Jabson and Kamen, 2016); therefore, an intervention that is able to reach them remotely, outside of a clinical setting, may be especially valuable. Further, LGBTQ+ couples face specific and unique RSH needs that may not be encompassed in current interventions (Brown and McElroy, 2018; Damaskos et al., 2018; Boehmer et al., 2020). Therefore, *Opening the Conversation* aims to provide inclusive informational resources as well as the flexibility to focus on couples’ unique RSH needs.

Participants emphasized that it was essential to provide education and support for both partners as part of the adapted intervention, noting a distinct lack of support for those partners without a cancer history. Research demonstrates that partners of cancer survivors experience unique needs that often remain unaddressed by cancer support services, and existing interventions for partners are rarely implemented in practice (Northouse et al., 2012). In formative research with the intended audience, couples also stressed the need for partner-specific resources, such as support groups, informational resources, and skill building to support survivors (Gorman et al., 2020). Survivors and their partners indicated a specific need for practical/problem-oriented support, such as transportation to appointments, involvement of partners during appointments, and emotional support, which is exemplified by the development of skills to comfort the survivor during difficult decisions and ability to express physical intimacy (e.g., gentle touch and hugs; Gorman et al., 2020). Therefore, intervention modifications included the addition of resources and educational information for both partners and underscored the importance of both partners reviewing and discussing materials together.

Strengths of the study included triangulation of decisions across multiple stakeholder perspectives to make adaptation decisions, purposeful inclusion of LGBTQ+ perspectives, and an iterative, systematic process of intervention adaptation with the goal of optimizing feasibility and acceptability. While we could not achieve data saturation with the small number of LGBTQ+ participants, we gained critical insight on the needs and preferences of this population from survivors and partners, including from community advisors at multiple time points. The emphasis on inclusion is important given the current lack of supportive care resources for LGBTQ+ survivors, partners, and couples (Hill and Holborn, 2015; Brown and McElroy, 2018; Kamen et al., 2019). Another limitation is the specific focus on breast and gynecologic cancer survivors; young couples with other types of cancer also experience RSH concerns (Karabulut and Erci, 2009; Schover et al., 2014; Ljungman et al., 2019). Finally, although we sought broad inclusion of stakeholders and cancer survivor couples throughout the adaptation process, the sample is small and mostly identified as White and college educated. Because adaptations reflect the experiences and perspectives of our sample of participants and stakeholders, adaptations may not generalize to a broader audience of young survivor couples. If this intervention proves effective, future research could adapt the intervention further to meet the needs of other audiences.

Addressing the RSH concerns of young adult breast and gynecologic cancer survivors and their partners is essential, and supportive care interventions are scarce. This study yielded a novel and inclusive dyadic coping and communication intervention that can be tailored to help couples communicate about and cope with their current RSH concerns. Education and skills gained are expected to support couples in addressing new concerns that may arise after the conclusion of the intervention. *Opening the Conversation* will be evaluated in a randomized controlled trial, with the long-term goal of broad implementation and dissemination as part of a comprehensive, coordinated survivorship care strategy for young adult couples with diverse backgrounds who are experiencing RSH concerns. In the intervention proves effective, future research will to explore implementation strategies in cancer care settings.

## Data Availability Statement

The datasets presented in this article are not readily available because of the sensitive nature of the research and confidentiality concerns; participants did not consent to data sharing. Requests to access the datasets should be directed to JG, Jessica.Gorman@oregonstate.edu.

## Ethics Statement

The studies involving human participants were reviewed and approved by the Oregon State University Institutional Review Board. The patients/participants provided their verbal informed consent to participate in this study.

## Author Contributions

JG, KL, JR, SH, and CA: conceptualization. JG: methodology, writing—original, and funding acquisition. LF, KL, JR, CA, JS, and BH-L: validation. JG, ES, and JD: formal analysis and investigation. JG, KL, JR, CA, ES, JD, JS, LF, BH-L, and SH: writing—reviewing and editing. BH-L and JS: supervision. All authors contributed to the article and approved the submitted version.

## Funding

This study was funded by the American Cancer Society (RSG-19-123-01-CPPB).

## Conflict of Interest

The authors declare that the research was conducted in the absence of any commercial or financial relationships that could be construed as a potential conflict of interest.

## Publisher’s Note

All claims expressed in this article are solely those of the authors and do not necessarily represent those of their affiliated organizations, or those of the publisher, the editors and the reviewers. Any product that may be evaluated in this article, or claim that may be made by its manufacturer, is not guaranteed or endorsed by the publisher.

## References

[ref1] Acquati C. Kayser K. (2019). Dyadic coping Across the lifespan: A comparison Between younger and middle-aged couples With breast cancer. Front. Psychol. 10:404. doi: 10.3389/fpsyg.2019.00404, PMID: 30941068 PMC6433932

[ref2] Arndt V. Merx H. Sturmer T. Stegmaier C. Ziegler H. Brenner H. (2004). Age-specific detriments to quality of life among breast cancer patients one year after diagnosis. Eur. J. Cancer 40, 673–680. doi: 10.1016/j.ejca.2003.12.007, PMID: 15010067

[ref3] Badr H. (2017). New frontiers in couple-based interventions in cancer care: refining the prescription for spousal communication. Acta Oncol. 56, 139–145. doi: 10.1080/0284186X.2016.1266079, PMID: 27937437

[ref4] Badr H. Acitelli L. K. Taylor C. L. (2008). Does talking about their relationship affect couples' marital and psychological adjustment to lung cancer? J. Cancer Surviv. 2, 53–64. doi: 10.1007/s11764-008-0044-3, PMID: 18648987

[ref5] Badr H. Krebs P. (2013). A systematic review and meta-analysis of psychosocial interventions for couples coping with cancer. Psychooncology 22, 1688–1704. doi: 10.1002/pon.3200, PMID: 23045191 PMC3562417

[ref6] Badr H. Taylor C. L. (2009). Sexual dysfunction and spousal communication in couples coping with prostate cancer. Psychooncology 18, 735–746. doi: 10.1002/pon.1449, PMID: 19061199 PMC4476400

[ref8] Benyamini Y. Gozlan M. Kokia E. (2009). Women's and men's perceptions of infertility and their associations with psychological adjustment: a dyadic approach. Br. J. Health Psychol. 14, 1–16. doi: 10.1348/135910708X279288, PMID: 18230194

[ref9] Bidstrup P. E. Christensen J. Mertz B. G. Rottmann N. Dalton S. O. Johansen C. (2015). Trajectories of distress, anxiety, and depression among women with breast cancer: looking beyond the mean. Acta Oncol. 54, 789–796. doi: 10.3109/0284186X.2014.1002571, PMID: 25761086

[ref10] Bodenmann G. (1995). A systemic-transactional conceptualization of stress and coping in couples. Swiss J. Psychol. 54, 34–49.

[ref11] Bodenmann G. (1997). Dyadic coping: A systemic-transactional view of stress and coping among couples: theory and empirical findings. Eur. Rev. Appl. Psychol. 47, 137–141.

[ref12] Bodenmann G. (2007). “Dyadic coping and the 3-phase-method in working with couples” in Innovations in Clinical Practice: Focus on Group and Family Therapy. ed. Vandecreek L. (Sarasota: Professional Resources Press), 235–252.

[ref13] Bodenmann G. (2008). Dyadic coping and the significance of this concept for prevention and therapy. Zeitschrift Gesundheitspsychol. 16, 108–111. doi: 10.1026/0943-8149.16.3.108

[ref14] Bodenmann G. Pihet S. Kayser K. (2006). The relationship between dyadic coping and marital quality: a 2-year longitudinal study. J. Fam. Psychol. 20, 485–493. doi: 10.1037/0893-3200.20.3.485, PMID: 16938007

[ref15] Bodenmann G. Randall A. K. Falconier M. K. (2016). Coping in Couples: The Systemic Transactional Model (STM), In Couples Coping with Stress: A Cross-Cultural Perspective. New York: Routledge/Taylor & Francis Group, 5–22.

[ref16] Bodenmann G. Shantinath S. D. (2004). The couples coping enhancement training (CCET): A new approach to prevention of marital distress based upon stress and coping. Fam. Relat. 53, 477–484. doi: 10.1111/j.0197-6664.2004.00056.x

[ref17] Boehmer U. Glickman M. Winter M. Clark M. A. (2013). Long-term breast cancer survivors' symptoms and morbidity: differences by sexual orientation? J. Cancer Surviv. 7, 203–210. doi: 10.1007/s11764-012-0260-8, PMID: 23328868

[ref18] Boehmer U. Stokes J. E. Bazzi A. R. Clark M. A. (2020). Dyadic quality of life among heterosexual and sexual minority breast cancer survivors and their caregivers. Support. Care Cancer 28, 2769–2778. doi: 10.1007/s00520-019-05148-7, PMID: 31724075 PMC7183428

[ref19] Bradford A. Fellman B. Urbauer D. Gallegos J. Meaders K. Tung C.. (2015). Assessment of sexual activity and dysfunction in medically underserved women with gynecologic cancers. Gynecol. Oncol. 139, 134–140. doi: 10.1016/j.ygyno.2015.08.019, PMID: 26325527 PMC4587341

[ref20] Braun V. Clarke V. (2006). Using thematic analysis in psychology. Qual. Res. Psychol. 3, 77–101. doi: 10.1191/1478088706qp063oa

[ref21] Brown M. T. Mcelroy J. A. (2018). Unmet support needs of sexual and gender minority breast cancer survivors. Support. Care Cancer 26, 1189–1196. doi: 10.1007/s00520-017-3941-z, PMID: 29080921

[ref22] Canada A. L. Schover L. R. (2012). The psychosocial impact of interrupted childbearing in long-term female cancer survivors. Psychooncology 21, 134–143. doi: 10.1002/pon.1875, PMID: 22271533 PMC3123665

[ref23] Carroll A. J. Baron S. R. Carroll R. A. (2016). Couple-based treatment for sexual problems following breast cancer: A review and synthesis of the literature. Support. Care Cancer 24, 3651–3659. doi: 10.1007/s00520-016-3218-y, PMID: 27154014

[ref24] Carter J. Chi D. S. Brown C. L. Abu-Rustum N. R. Sonoda Y. Aghajanian C.. (2010). Cancer-related infertility in survivorship. Int. J. Gynecol. Cancer 20, 2–8. doi: 10.1111/IGC.0b013e3181bf7d3f, PMID: 20130497

[ref25] Damaskos P. Amaya B. Gordon R. Walters C. B. (2018). Intersectionality and the LGBT cancer patient. Semin. Oncol. Nurs. 34, 30–36. doi: 10.1016/j.soncn.2017.11.004, PMID: 29325815 PMC7424551

[ref26] Dobinson K. A. Hoyt M. A. Seidler Z. E. Beaumont A. L. Hullmann S. E. Lawsin C. R. (2015). A grounded theory investigation into the psychosexual unmet needs of adolescent and young adult cancer survivors. J. Adolesc. Young Adult Oncol. 5, 135–145. doi: 10.1089/jayao.2015.0022 26812456

[ref27] Dryden A. Ussher J. M. Perz J. (2014). Young women's construction of their post-cancer fertility. Psychol. Health 29, 1341–1360. doi: 10.1080/08870446.2014.932790, PMID: 24916140

[ref28] Epstein N. B. Baucom D. H. (2002). Enhanced Cognitive-Behavioral Therapy for Couples: A Contextual Approach. Washington, DC: American Psychological Association.

[ref29] Escoffery C. Lebow-Skelley E. Udelson H. Boing E. A. Wood R. Fernandez M. E.. (2019). A scoping study of frameworks for adapting public health evidence-based interventions. Transl. Behav. Med. 9, 1–10. doi: 10.1093/tbm/ibx067, PMID: 29346635 PMC6305563

[ref30] Fleishman J. M. Crane B. Koch P. B. (2020). Correlates and predictors of sexual satisfaction for older adults in same-sex relationships. J. Homosex. 67, 1974–1998. doi: 10.1080/00918369.2019.1618647, PMID: 31172878

[ref31] Fobair P. Spiegel D. (2009). Concerns about sexuality after breast cancer. Cancer J. 15, 19–26. doi: 10.1097/PPO.0b013e31819587bb 19197169

[ref32] Fobair P. Stewart S. L. Chang S. B. D'onofrio C. Banks P. J. Bloom J. R. (2006). Body image and sexual problems in young women with breast cancer. Psychooncology 15, 579–594. doi: 10.1002/pon.991, PMID: 16287197

[ref33] Fredman S. J. Baucom D. H. Gremore T. M. Castellani A. M. Kallman T. A. Porter L. S.. (2009). Quantifying the recruitment challenges with couple-based interventions for cancer: applications to early-stage breast cancer. Psychooncology 18, 667–673. doi: 10.1002/pon.1477, PMID: 19061201 PMC4506748

[ref34] Ganz P. A. Desmond K. A. Leedham B. Rowland J. H. Meyerowitz B. E. Belin T. R. (2002). Quality of life in long-term, disease-free survivors of breast cancer: a follow-up study. J. Natl. Cancer Inst. 94, 39–49. doi: 10.1093/jnci/94.1.39, PMID: 11773281

[ref35] Gilbert E. Ussher J. M. Perz J. (2011). Sexuality after gynaecological cancer: a review of the material, intrapsychic, and discursive aspects of treatment on women's sexual-wellbeing. Maturitas 70, 42–57. doi: 10.1016/j.maturitas.2011.06.013, PMID: 21764229

[ref36] Gorman J. R. Bailey S. Pierce J. P. Su H. I. (2012). How do you feel about fertility and parenthood? The voices of young female cancer survivors. J. Cancer Surviv. 6, 200–209. doi: 10.1007/s11764-011-0211-9, PMID: 22179785 PMC3667153

[ref37] Gorman J. R. Drizin J. H. Smith E. Flores-Sanchez Y. Harvey S. M. (2021). Patient-centered communication to address young adult breast cancer Survivors' reproductive and sexual health concerns. Health Commun. 36, 1743–1758. doi: 10.1080/10410236.2020.1794550, PMID: 32703034

[ref38] Gorman J. R. Smith E. Drizin J. H. Lyons K. S. Harvey S. M. (2020). Navigating sexual health in cancer survivorship: a dyadic perspective. Support. Care Cancer 28, 5429–5439. doi: 10.1007/s00520-020-05396-y, PMID: 32157507

[ref39] Gorman J. R. Su H. I. Pierce J. P. Roberts S. C. Dominick S. A. Malcarne V. L. (2014). A multidimensional scale to measure the reproductive concerns of young adult female cancer survivors. J. Cancer Surviv. 8, 218–228. doi: 10.1007/s11764-013-0333-3, PMID: 24352870 PMC4016119

[ref40] Gorman J. R. Usita P. M. Madlensky L. Pierce J. P. (2011). Young breast cancer survivors: their perspectives on treatment decisions and fertility concerns. Cancer Nurs. 34, 32–40. doi: 10.1097/NCC.0b013e3181e4528d, PMID: 20697269 PMC2980796

[ref41] Grover S. Hill-Kayser C. E. Vachani C. Hampshire M. K. Dilullo G. A. Metz J. M. (2012). Patient reported late effects of gynecological cancer treatment. Gynecol. Oncol. 124, 399–403. doi: 10.1016/j.ygyno.2011.11.034 22119992

[ref42] Hawkey A. Ussher J. M. Perz J. Parton C. (2021a). Talking but not always understanding: couple communication about infertility concerns after cancer. BMC Public Health 21:161. doi: 10.1186/s12889-021-10188-y, PMID: 33468106 PMC7816453

[ref43] Hawkey A. J. Ussher J. M. Perz J. Parton C. Patterson P. Bateson D.. (2021b). The impact of cancer-related fertility concerns on current and future couple relationships: people with cancer and partner perspectives. Eur. J. Cancer Care 30:e13348. doi: 10.1111/ecc.13348 33084134

[ref44] Hawkins Y. Ussher J. Gilbert E. Perz J. Sandoval M. Sundquist K. (2009). Changes in sexuality and intimacy after the diagnosis and treatment of cancer: the experience of partners in a sexual relationship with a person with cancer. Cancer Nurs. 32, 271–280. doi: 10.1097/NCC.0b013e31819b5a93, PMID: 19444088

[ref45] Heinrichs N. Zimmermann T. Huber B. Herschbach P. Russell D. W. Baucom D. H. (2012). Cancer distress reduction with a couple-based skills training: a randomized controlled trial. Ann. Behav. Med. 43, 239–252. doi: 10.1007/s12160-011-9314-9, PMID: 22037965

[ref46] Henderson A. W. Lehavot K. Simoni J. M. (2009). Ecological models of sexual satisfaction among lesbian/bisexual and heterosexual women. Arch. Sex. Behav. 38, 50–65. doi: 10.1007/s10508-008-9384-3, PMID: 18574685 PMC4083469

[ref47] Hill G. Holborn C. (2015). Sexual minority experiences of cancer care: A systematic review. J. Cancer Policy 6, 11–22. doi: 10.1016/j.jcpo.2015.08.005

[ref48] Hulbert-Williams N. J. Plumpton C. O. Flowers P. Mchugh R. Neal R. D. Semlyen J.. (2017). The cancer care experiences of gay, lesbian and bisexual patients: A secondary analysis of data from the UK cancer patient experience survey. Eur. J. Cancer Care 26:e12670. doi: 10.1111/ecc.12670, PMID: 28239936

[ref49] Hummel S. B. Hahn D. E. E. Van Lankveld J. Oldenburg H. S. A. Broomans E. Aaronson N. K. (2017a). Factors associated With specific diagnostic and statistical manual of mental disorders, fourth edition sexual dysfunctions in breast cancer survivors: A study of patients and their partners. J. Sex. Med. 14, 1248–1259. doi: 10.1016/j.jsxm.2017.08.004, PMID: 28923310

[ref50] Hummel S. B. Van Lankveld J. J. D. M. Oldenburg H. S. A. Hahn D. E. E. Kieffer J. M. Gerritsma M. A.. (2017b). Efficacy of internet-based cognitive behavioral therapy in improving sexual functioning of breast cancer survivors: results of a randomized controlled trial. J. Clin. Oncol. 35, 1328–1340. doi: 10.1200/JCO.2016.69.6021, PMID: 28240966

[ref51] Jabson J. M. Kamen C. S. (2016). Sexual minority cancer survivors' satisfaction with care. J. Psychosoc. Oncol. 34, 28–38. doi: 10.1080/07347332.2015.1118717, PMID: 26577277 PMC4916952

[ref52] Jing L. Zhang C. Li W. Jin F. Wang A. (2019). Incidence and severity of sexual dysfunction among women with breast cancer: a meta-analysis based on female sexual function index. Support. Care Cancer 27, 1171–1180. doi: 10.1007/s00520-019-04667-7, PMID: 30712099

[ref53] Kamen C. S. Alpert A. Margolies L. Griggs J. J. Darbes L. Smith-Stoner M.. (2019). "treat us with dignity": a qualitative study of the experiences and recommendations of lesbian, gay, bisexual, transgender, and queer (LGBTQ) patients with cancer. Support. Care Cancer 27, 2525–2532. doi: 10.1007/s00520-018-4535-0, PMID: 30411237 PMC6506401

[ref54] Kamen C. Mustian K. M. Dozier A. Bowen D. J. Li Y. (2015). Disparities in psychological distress impacting lesbian, gay, bisexual and transgender cancer survivors. Psychooncology 24, 1384–1391. doi: 10.1002/pon.3746, PMID: 25630987 PMC4517981

[ref55] Karabulut N. Erci B. (2009). Sexual desire and satisfaction in sexual life affecting factors in breast cancer survivors after mastectomy. J. Psychosoc. Oncol. 27, 332–343. doi: 10.1080/07347330902979101, PMID: 19544180

[ref56] Kayser K. Acquati C. Reese J. B. Mark K. Wittmann D. Karam E. (2018). A systematic review of dyadic studies examining relationship quality in couples facing colorectal cancer together. Psychooncology 27, 13–21. doi: 10.1002/pon.4339, PMID: 27943551

[ref57] Kayser K. Watson L. E. Adnrade J. T. (2007). Cancer as a "we-disease": examining the process of coping from a relational perspective. Fam. Syst. Health 25, 404–418. doi: 10.1037/1091-7527.25.4.404

[ref58] Keesing S. Rosenwax L. Mcnamara B. (2016). A dyadic approach to understanding the impact of breast cancer on relationships between partners during early survivorship. BMC Womens Health 16:57. doi: 10.1186/s12905-016-0337-z, PMID: 27561256 PMC5000504

[ref59] Kent E. E. Wheldon C. W. Smith A. W. Srinivasan S. Geiger A. M. (2019). Care delivery, patient experiences, and health outcomes among sexual and gender minority patients with cancer and survivors: A scoping review. Cancer 125, 4371–4379. doi: 10.1002/cncr.32388, PMID: 31593319

[ref60] Latham T. P. Sales J. M. Boyce L. S. Renfro T. L. Wingood G. M. Diclemente R. J.. (2010). Application of ADAPT-ITT: adapting an evidence-based HIV prevention intervention for incarcerated African American adolescent females. Health Promot. Pract. 11, 53S–60S. doi: 10.1177/1524839910361433, PMID: 20488969

[ref61] Lehmann V. Ferrante A. C. Winning A. M. Gerhardt C. A. (2019). The perceived impact of infertility on romantic relationships and singlehood among adult survivors of childhood cancer. Psychooncology 28, 622–628. doi: 10.1002/pon.4999, PMID: 30664284

[ref62] Lehmann V. Nahata L. Ferrante A. C. Hansen-Moore J. A. Yeager N. D. Klosky J. L.. (2018). Fertility-related perceptions and impact on romantic relationships among adult survivors of childhood cancer. J. Adolesc. Young Adult Oncol. 7, 409–414. doi: 10.1089/jayao.2017.0121, PMID: 29466084 PMC6083209

[ref63] Levin A. O. Carpenter K. M. Fowler J. M. Brothers B. M. Andersen B. L. Maxwell G. L. (2010). Sexual morbidity associated with poorer psychological adjustment among gynecological cancer survivors. Int. J. Gynecol. Cancer 20, 461–470. doi: 10.1111/IGC.0b013e3181d24ce0, PMID: 20375814 PMC3869624

[ref64] Li M. Chan C. W. H. Chow K. M. Xiao J. Choi K. C. (2020). A systematic review and meta-analysis of couple-based intervention on sexuality and the quality of life of cancer patients and their partners. Support. Care Cancer 28, 1607–1630. doi: 10.1007/s00520-019-05215-z, PMID: 31872299

[ref65] Ljungman L. Ahlgren J. Petersson L. M. Flynn K. E. Weinfurt K. Gorman J. R.. (2018). Sexual dysfunction and reproductive concerns in young women with breast cancer: type, prevalence, and predictors of problems. Psychooncology 27, 2770–2777. doi: 10.1002/pon.4886, PMID: 30203884 PMC6585728

[ref66] Ljungman L. Eriksson L. E. Flynn K. E. Gorman J. R. Stahl O. Weinfurt K.. (2019). Sexual dysfunction and reproductive concerns in young men diagnosed with testicular cancer: an observational study. J. Sex. Med. 16, 1049–1059. doi: 10.1016/j.jsxm.2019.05.005, PMID: 31255211

[ref67] Loaring J. M. Larkin M. Shaw R. Flowers P. (2015). Renegotiating sexual intimacy in the context of altered embodiment: the experiences of women with breast cancer and their male partners following mastectomy and reconstruction. Health Psychol. 34, 426–436. doi: 10.1037/hea0000195, PMID: 25822057

[ref68] Logan S. Perz J. Ussher J. M. Peate M. Anazodo A. (2018). A systematic review of patient oncofertility support needs in reproductive cancer patients aged 14 to 45 years of age. Psychooncology 27, 401–409. doi: 10.1002/pon.4502, PMID: 28734119

[ref69] Logan S. Perz J. Ussher J. M. Peate M. Anazodo A. (2019). Systematic review of fertility-related psychological distress in cancer patients: informing on an improved model of care. Psychooncology 28, 22–30. doi: 10.1002/pon.4927, PMID: 30460732

[ref70] Luk B. H. K. Loke A. Y. (2019). Sexual satisfaction, intimacy and relationship of couples undergoing infertility treatment. J. Reprod. Infant Psychol. 37, 108–122. doi: 10.1080/02646838.2018.1529407, PMID: 30317866

[ref71] Lyons K. S. Lee C. S. (2018). The theory of dyadic illness management. J. Fam. Nurs. 24, 8–28. doi: 10.1177/1074840717745669, PMID: 29353528

[ref72] Lyons K. S. Lee C. S. Bennett J. A. Nail L. M. Fromme E. K. Hiatt S. O.. (2014). Symptom incongruence trajectories in lung cancer dyads. J. Pain Symptom Manag. 48, 1031–1040. doi: 10.1016/j.jpainsymman.2014.02.004, PMID: 24747222

[ref73] Lyons K. S. Miller L. M. Mccarthy M. J. (2016). The roles of dyadic appraisal and dyadic coping in couples with lung cancer. J. Fam. Nurs. 22, 493–514. doi: 10.1177/1074840716675976, PMID: 27803239 PMC5405735

[ref74] Manne S. Badr H. (2008). Intimacy and relationship processes in couples' psychosocial adaptation to cancer. Cancer 112, 2541–2555. doi: 10.1002/cncr.23450, PMID: 18428202 PMC4449141

[ref75] Manne S. L. Kissane D. Zaider T. Kashy D. Lee D. Heckman C.. (2015). Holding back, intimacy, and psychological and relationship outcomes among couples coping with prostate cancer. J. Fam. Psychol. 29, 708–719. doi: 10.1037/fam0000096, PMID: 26192132 PMC5225663

[ref76] Manne S. L. Ostroff J. S. Norton T. R. Fox K. Goldstein L. Grana G. (2006). Cancer-related relationship communication in couples coping with early stage breast cancer. Psychooncology 15, 234–247. doi: 10.1002/pon.941, PMID: 15926198

[ref77] Mckleroy V. S. Galbraith J. S. Cummings B. Jones P. Harshbarger C. Collins C.. (2006). Adapting evidence-based behavioral interventions for new settings and target populations. AIDS Educ. Prev. 18, 59–73. doi: 10.1521/aeap.2006.18.supp.59, PMID: 16987089

[ref78] Northouse L. Williams A. L. Given B. Mccorkle R. (2012). Psychosocial care for family caregivers of patients with cancer. J. Clin. Oncol. 30, 1227–1234. doi: 10.1200/JCO.2011.39.5798 22412124

[ref79] Otto A. K. Ketcher D. Heyman R. E. Vadaparampil S. T. Ellington L. Reblin M. (2021). Communication between advanced cancer patients and their family caregivers: relationship with caregiver burden and preparedness for caregiving. Health Commun. 36, 714–721. doi: 10.1080/10410236.2020.1712039, PMID: 31910681 PMC9118123

[ref80] Palinkas L. A. Horwitz S. M. Green C. A. Wisdom J. P. Duan N. Hoagwood K. (2015). Purposeful sampling for qualitative data collection and analysis in mixed method implementation research. Admin. Pol. Ment. Health 42, 533–544. doi: 10.1007/s10488-013-0528-y, PMID: 24193818 PMC4012002

[ref81] Patterson P. Perz J. Tindle R. Mcdonald F. E. J. Ussher J. M. (2020). Infertility after cancer: how the need to be a parent, fertility-related social concern, and acceptance of illness influence quality of life. Cancer Nurs. 44, E244–E251. doi: 10.1097/ncc.0000000000000811 32209862

[ref82] Perz J. Ussher J. M. Gilbert E. Australian C. Sexuality Study, T (2014). Feeling well and talking about sex: psycho-social predictors of sexual functioning after cancer. BMC Cancer 14:228. doi: 10.1186/1471-2407-14-228 24673768 PMC3986691

[ref7] Reese J. B. Porter L. S. Regan K. R. Keefe F. J. Azad N. S. Diaz L. A. Jr.. (2014). A randomized pilot trial of a telephone-based couples intervention for physical intimacy and sexual concerns in colorectal cancer. Psycho-Oncology 23, 1005–1013. doi: 10.1002/pon.3508, PMID: 24615831 PMC4618456

[ref83] Reese J. B. Porter L. S. Casale K. E. Bantug E. T. Bober S. L. Schwartz S. C.. (2016). Adapting a couple-based intimacy enhancement intervention to breast cancer: A developmental study. Health Psychol. 35, 1085–1096. doi: 10.1037/hea0000413, PMID: 27657981 PMC5034713

[ref84] Reese J. B. Smith K. C. Handorf E. Sorice K. Bober S. L. Bantug E. T.. (2019). A randomized pilot trial of a couple-based intervention addressing sexual concerns for breast cancer survivors. J. Psychosoc. Oncol. 37, 242–263. doi: 10.1080/07347332.2018.1510869, PMID: 30580675 PMC6476670

[ref85] Regan T. W. Lambert S. D. Girgis A. Kelly B. Kayser K. Turner J. (2012). Do couple-based interventions make a difference for couples affected by cancer? A systematic review. BMC Cancer 12:279. doi: 10.1186/1471-2407-12-279, PMID: 22769228 PMC3464780

[ref86] Robertson E. G. Sansom-Daly U. M. Wakefield C. E. Ellis S. J. Mcgill B. C. Doolan E. L.. (2016). Sexual and romantic relationships: experiences of adolescent and young adult cancer survivors. J. Adolesc. Young Adult Oncol. 5, 286–291. doi: 10.1089/jayao.2015.0061, PMID: 26885746

[ref87] Robinson L. Miedema B. Easley J. (2014). Young adult cancer survivors and the challenges of intimacy. J. Psychosoc. Oncol. 32, 447–462. doi: 10.1080/07347332.2014.917138, PMID: 24797721

[ref88] Saldaña J. (2015). The Coding Manual for Qualitative Researchers. London: SAGE Publications

[ref89] Schover L. R. Van Der Kaaij M. Van Dorst E. Creutzberg C. Huyghe E. Kiserud C. E. (2014). Sexual dysfunction and infertility as late effects of cancer treatment. EJC Suppl. 12, 41–53. doi: 10.1016/j.ejcsup.2014.03.004, PMID: 26217165 PMC4250536

[ref90] Schover L. R. Yuan Y. Fellman B. M. Odensky E. Lewis P. E. Martinetti P. (2013). Efficacy trial of an internet-based intervention for cancer-related female sexual dysfunction. J. Natl. Compr. Cancer Netw. 11, 1389–1397. doi: 10.6004/jnccn.2013.0162, PMID: 24225972 PMC3831175

[ref91] Scott J. L. Halford W. K. Ward B. G. (2004). United we stand? The effects of a couple-coping intervention on adjustment to early stage breast or gynecological cancer. J. Consult. Clin. Psychol. 72, 1122–1135. doi: 10.1037/0022-006X.72.6.1122 15612858

[ref92] Scott J. L. Kayser K. (2009). A review of couple-based interventions for enhancing women's sexual adjustment and body image after cancer. Cancer J. 15, 48–56. doi: 10.1097/PPO.0b013e31819585df, PMID: 19197174

[ref93] Seay J. Mitteldorf D. Yankie A. Pirl W. F. Kobetz E. Schlumbrecht M. (2018). Survivorship care needs among LGBT cancer survivors. J. Psychosoc. Oncol. 36, 393–405. doi: 10.1080/07347332.2018.1447528, PMID: 29791273

[ref94] Stabile C. Goldfarb S. Baser R. E. Goldfrank D. J. Abu-Rustum N. R. Barakat R. R.. (2017). Sexual health needs and educational intervention preferences for women with cancer. Breast Cancer Res. Treat. 165, 77–84. doi: 10.1007/s10549-017-4305-6, PMID: 28547655 PMC5515493

[ref95] Traa M. J. De Vries J. Bodenmann G. Den Oudsten B. L. (2015). Dyadic coping and relationship functioning in couples coping with cancer: a systematic review. Br. J. Health Psychol. 20, 85–114. doi: 10.1111/bjhp.12094, PMID: 24628822

[ref96] Ussher J. M. Perz J. Gilbert E. (2014). Women's sexuality after cancer: A qualitative analysis of sexual changes and renegotiation. Women Ther. 37, 205–221. doi: 10.1080/02703149.2014.897547

[ref97] Vaz A. F. Pinto-Neto A. M. Conde D. M. Costa-Paiva L. Morais S. S. Pedro A. O.. (2011). Quality of life and menopausal and sexual symptoms in gynecologic cancer survivors: a cohort study. Menopause 18, 662–669. doi: 10.1097/gme.0b013e3181ffde7f, PMID: 21471827

[ref98] Walshe J. M. Denduluri N. Swain S. M. (2006). Amenorrhea in premenopausal women after adjuvant chemotherapy for breast cancer. J. Clin. Oncol. 24, 5769–5779. doi: 10.1200/JCO.2006.07.2793 17130515

[ref99] Wettergren L. Kent E. E. Mitchell S. A. Zebrack B. Lynch C. F. Rubenstein M. B.. (2017). Cancer negatively impacts on sexual function in adolescents and young adults: The AYA HOPE study. Psychooncology 26, 1632–1639. doi: 10.1002/pon.4181 27240019 PMC7239373

[ref100] Wiltsey Stirman S. Baumann A. A. Miller C. J. (2019). The FRAME: an expanded framework for reporting adaptations and modifications to evidence-based interventions. Implement. Sci. 14:58. doi: 10.1186/s13012-019-0898-y, PMID: 31171014 PMC6554895

[ref101] Wingood G. M. Diclemente R. J. (2008). The ADAPT-ITT model: a novel method of adapting evidence-based HIV interventions. J. Acquir. Immune Defic. Syndr. 47, S40–S46. doi: 10.1097/QAI.0b013e3181605df1, PMID: 18301133

[ref102] Zimmermann T. Heinrichs N. Scott J. L. (2006). CanCOPE "Schritt für Schritt": die Effektivität eines partnerschaftlichen Unterstützungsprogramms bei frauen mit Brust- oder gynäkologischen Krebserkrankungen. [CanCOPE 'Step by Step': The effectiveness of a couple-based intervention program for women with breast or gynecological cancer]. Verhaltenstherapie 16, 247–255. doi: 10.1159/000096122

